# The Comparative Validity of Interactive Multimedia Questionnaires to Paper-Administered Questionnaires for Beverage Intake and Physical Activity: Pilot Study

**DOI:** 10.2196/resprot.2830

**Published:** 2013-10-22

**Authors:** Shaun K Riebl, Allyson C Paone, Valisa E Hedrick, Jamie M Zoellner, Paul A Estabrooks, Brenda M Davy

**Affiliations:** ^1^Virginia TechDepartment of Human Nutrition, Foods, and ExerciseBlacksburg, VAUnited States

**Keywords:** validity and reliability, multimedia, dietary assessment, beverages, physical activity

## Abstract

**Background:**

Brief, valid, and reliable dietary and physical activity assessment tools are needed, and interactive computerized assessments (ie, those with visual cues, pictures, sounds, and voiceovers) can reduce administration and scoring burdens commonly encountered with paper-based assessments.

**Objective:**

The purpose of this pilot investigation was to evaluate the comparative validity and reliability of interactive multimedia (IMM) versions (ie, IMM-1 and IMM-2) compared to validated paper-administered (PP) versions of the beverage intake questionnaire (BEVQ-15) and Stanford Leisure-Time Activity Categorical Item (L-Cat); a secondary purpose was to evaluate results across two education attainment levels.

**Methods:**

Adults 21 years or older (n=60) were recruited to complete three laboratory sessions, separated by three to seven days in a randomly assigned sequence, with the following assessments–demographic information, two IMM and one paper-based (PP) version of the BEVQ-15 and L-Cat, health literacy, and an IMM usability survey.

**Results:**

Responses across beverage categories from the IMM-1 and PP versions (validity; *r*=.34-.98) and the IMM-1 and IMM-2 administrations (reliability; *r*=.61-.94) (all *P*<.001) were significantly correlated. Paired *t* tests revealed significant differences in sugar-sweetened beverage (SSB) grams and kcal (*P*=.02 and *P*=.01, respectively) and total beverage kcal (*P*=.03), on IMM-1 and IMM-2; however, comparative validity was demonstrated between IMM-2 and the PP version suggesting familiarization with the IMM tool may influence participant responses (mean differences: SSB 63 grams, SEM 87; *P*=.52; SSB 21 kcal, SEM 33; *P*=.48; total beverage 65 kcal, SEM 49; *P*=.19). Overall mean scores between the PP and both IMM versions of the L-Cat were different (both *P*<.001); however, responses on all versions were correlated (*P*<.001). Differences between education categories were noted at each L-Cat administration (IMM-1: *P*=.008; IMM-2: *P*=.001; PP: *P*=.002). Major and minor themes from user feedback suggest that the IMM questionnaires were easy to complete, and relevant to participants' typical beverage choices and physical activity habits.

**Conclusions:**

In general, less educated participants consumed more total beverage and SSB energy, and reported less engagement in physical activity. The IMM BEVQ-15 appears to be a valid and reliable measure to assess habitual beverage intake, although software familiarization may increase response accuracy. The IMM-L-Cat can be considered reliable and may have permitted respondents to more freely disclose actual physical activity levels versus the paper-administered tool. Future larger-scale investigations are warranted to confirm these possibilities.

##  Introduction

### Assessment Methods

Multiple unannounced 24-hour recalls, food records, and physical activity recalls have historically been recognized as gold standard approaches to assessing dietary and physical activity behaviors [1-3]. However, these assessment methods often require trained staff to administer and analyze, and are labor-intensive for both researchers and participants [1,2]. For these reasons, other valid methods of diet and physical activity assessments have been developed, such as food-frequency questionnaires as well as brief assessments of diet and physical activity. Recently, computerized diet and physical activity assessments have emerged as a way to decrease literacy barriers for participants, as well as decrease the research burden of processing paper-based surveys in large studies [4-8]. However, attention to the reliability, validity, and usability of computerized approaches to assessing diet and physical activity behaviors are imperative.

Due in part to the increased use and accessibility of computers in multiple settings (eg, homes, libraries, churches, recreational community centers, grocery stores, and schools) [7,9], the use of Web and computer-based assessments in large research trials have increased over the past 10 years [4,6,10]. The National Institutes of Health has recognized the need for novel/innovative assessment methods using technological advances in physical activity and dietary assessment (eg, PAR-12-198). There is no consensus to whether a paper-based assessment is superior to a computerized one [11]; however, computer-based tools can provide an alternative means to collect and analyze data [12] and may be appealing to practitioners and researchers because of their proposed benefits. Computer-administered assessments may overcome difficulties sometimes associated with paper-based surveys as they allow for interactivity-two-way communication between computer and participant through photographs, videos, and displayed text with or without audio [7]. Other advantages of computerized questionnaire administration include-complete responses (ie, prompting individuals to answer all questions), written and narrated text, visual cues of portion sizes, immediate and rapid data entry and scoring, decreased scoring errors, increased attentiveness from participants, instantaneous feedback, and a greater ability to access understudied populations [6,8,10,13,14]. Additionally, multi-part questions of computerized assessments can be programmed to reduce administration time by providing only relevant data and information for the participant [7]. In low health literacy populations, computerized questionnaires may be advantageous since text can be narrated and visual aids can be used, which may reduce response errors and the necessity of advanced reading skills [7]. Another potential advantage of computer-based assessments is that response-bias and intimidation may be reduced with computer-administered surveys, although additional research addressing this possibility is needed [5,7,15]. However, when using identical computerized versions of paper assessments comparability cannot be assumed because interface characteristics like font size, line length, scrolling ability, and amount of information visible on the screen can all influence user performance [16,17].

### Two Paper-Based Questionnaires

Prior research has demonstrated the reliability and validity of two self-administered paper-based questionnaires. One assesses habitual beverage intake (BEVQ-15) [18], and the other measures usual physical activity level, Stanford Leisure-Time Activity Categorical Item 2.2 (L-Cat) [19]. There are several computerized nutrition education delivery [7,20,21] and dietary assessment tools [8,13,22-28] and a few Web-based physical activity questionnaires [12,29,30] currently available; however, to the best of our knowledge, no computer-based beverage intake questionnaire exists. The recently developed Automated Self-Administered 24-hour Recall [31] is computer-based and does contain questions about beverage intake; however, results on its validity and usability have yet to be published [32]. The purpose of this pilot investigation was to evaluate the comparative validity and reliability of newly developed interactive multimedia (IMM) versions compared to validated paper-administered (PP) versions of the BEVQ-15 [18] and L-Cat [19]. Individuals with lower educational attainment and/or health literacy levels may be at increased risk for health complications associated with poor dietary and health behaviors such as obesity, diabetes, hypertension, and coronary heart disease [33]. Therefore, a secondary purpose of this investigation was to evaluate the validity and reliability of the major BEVQ-15 categories, for example, total water, sugar-sweetened beverage (SSB), and total beverage grams and kcal, and L-Cat category across two education levels, in order to determine the suitability of the IMM versions for individuals from varying educational backgrounds.

##  Methods

### Recruitment

Adults 21 years or older (n=60) were recruited from several community settings (a local university community, free medical clinic, area Community Services building, and church congregation) between January and August 2012 in southwest Virginia. The Virginia Tech Institutional Review Board approved the study protocol and participants provided written informed consent prior to enrollment.

### Protocol

Participation entailed three laboratory sessions with three to seven days between each session. Sessions were completed in one of two randomly assigned visit sequences that differed in questionnaire administration format (ie, taking the paper or computerized instruments initially). Randomization was done to avoid a potential effect of session order on study outcomes. In addition to providing demographic information, each participant completed a total of two identical self-administered IMM BEVQ-15 [18] and L-Cat [19] questionnaires (denoted IMM-1 for the first administration and IMM-2 for the second administration), one PP BEVQ-15 and L-Cat (ie, one set being BEVQ-15 and L-Cat at each of the three lab sessions), the Newest Vital Sign tool to assess health literacy [34], and an open-ended feedback survey on the IMM questionnaire to address usability; a total of five questionnaires were completed by each participant. Responses from the feedback survey were either "yes" or "no", or rated on an ordered-response scale (1=easy, 5=hard) with open-ended areas for comments following each question. Investigators supervised the assessments and provided limited instructions, but were available to answer questions during the survey if needed. Participants were compensated in the form of a $25 gift card upon completion of all three study visits.

### Measurements

Participants provided information on demographic characteristics (ie, age, race/ethnicity, income level, and highest education level attained), and this was used to categorize participants into one of two education categories: (1) less than high school/high school, and (2) some college/college degree. Prior research suggests that the level of education reached can be a strong socioeconomic determinant of beverage intake [33,35]. Descriptive measurements were conducted by a graduate research assistant who is a registered dietitian (SKR) and a trained research assistant (ACP) and included the following-height measured in meters without shoes using a wall mounted stadiometer (Seca 216, Hamburg, Germany); body weight measured in light clothing without shoes to the nearest 0.2 kg using a digital scale (Scale-Tronix, Wheaton, IL); and body mass index (BMI), calculated as kg/m^2^. The Newest Vital Sign (NVS) is a valid and sensitive tool that was used to assess health literacy and includes six questions based upon information contained in a nutrition facts label for a pint of ice cream. The scores range from 0-6 (0=limited health literacy, 6=adequate health literacy) [34].

### The BEVQ-15 and L-Cat

The BEVQ-15 [18] is a brief, valid, and reliable quantitative food frequency questionnaire providing an estimate of habitual beverage intake across 15 beverage categories, which evaluates total beverage and SSB intake (ie, grams and kcal) over the past 30 days. Details regarding the development and evaluation of the BEVQ-15 have been previously published [18,36]. The PP BEVQ took 2 minutes-15 seconds to complete during its initial testing [18]. Self-reported physical activity was assessed using the brief L-Cat [19]. This tool was developed from the Stanford Brief Activity Survey [37-39] and consists of six descriptive categories (eg, 3="About three times a week, I did moderate activities such as brisk walking, swimming, or riding a bike for about 15-20 minutes each time. Or about once a week, I did moderately difficult chores such as raking or mowing the lawn for about 45-60 minutes. Or about once a week, I played sports such as softball, basketball, or soccer for about 45-60 minutes.") ranging from inactive (1=I did not do much physical activity) to very active (6=Almost daily, that is five or more times a week, I did vigorous activities). In a randomized trial involving 267 obese women, the L-Cat was found to be valid and reliable [19]. The paper versions of these tools were read and completed by the study participants independently with an investigator available for questions throughout the administration.

**Figure 1:**
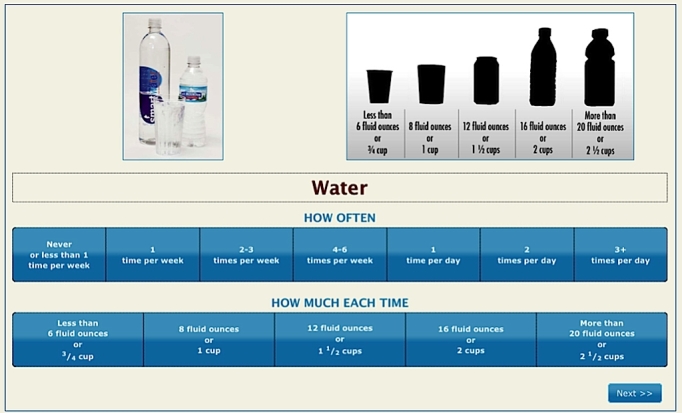
Screenshot example of the water beverage category from the interactive multimedia Beverage Intake Questionnaire-15.

Using the validated PP versions of these two tools, computerized versions were developed. The IMM BEVQ-15 began with narrated text and graphic on-screen directions taking approximately three to four minutes. For each drink category, which replicated the paper-based BEVQ-15 in content and sequence, a photo of the beverage was presented on-screen (Figure 1 shows an example of the water intake category). Silhouettes of portion sizes with the quantities they represented in fluid ounces and cups were presented for each beverage category. For example, a soft drink can silhouette was pictured with other beverage containers with the text stating, "a typical beverage can represents 12 fluid ounces or 1 ½ cups." Once the IMM BEVQ-15 was completed, the participant was directed to an instructional page with narrated text (approximately 25 seconds) describing the L-Cat. As with the IMM BEVQ-15, the IMM L-Cat provided audio for each category wherever the mouse's cursor was placed. When the participant chose the physical activity/leisure time activity statement that best reflected their usual physical activity level another completion page was displayed which informed the participant that they were through with the computerized assessment. Completion time was covertly monitored for the IMM BEVQ and L-Cat. After finishing the first IMM administration, participants were invited to complete a user feedback survey that contained seven questions with ordered-response (eg, 1=easy and 5= hard), "Yes" or "No," and open-end response formats.

### Statistical Analysis

Statistical analyses were performed using SPSS statistical analysis software (version 20.0 for Macintosh, 2011, IBM Corporation, Chicago, IL). Demographic characteristics and mean daily beverage consumption (grams, kcal) for the two IMM and one PP BEVQ are reported as mean and standard error of the mean (SEM) or as frequencies (categorical variables). Paired sample *t* tests and bivariate correlations (Pearson's *r*) were used to assess validity (first IMM administration vs PP) and reliability (first vs second IMM administration, or IMM-1 vs IMM-2). Due to multiple *t* tests being conducted, data were reanalyzed to evaluate major outcomes using repeated measures analysis of variance with covariates where appropriate (eg, education), and results were consistent across analytical approaches. Chi square analyses and independent sample *t* tests were used to assess differences between education groups on categorical variables (eg, gender, race/ethnicity) and continuous variables (eg, BMI, beverage consumption), respectively. The alpha level was set *a priori* at *P*≤.05. Responses from the open-ended ease of use questions on the IMM feedback survey were grouped into themes and quantified. *Major themes* were considered similar responses from ≥50% of participants (≥30 of 60 participants), while *minor themes* were considered similar responses from 25%-49% of participants (15 to 29, of 60 participants) [40]. A second investigator independently verified themes.

##  Results

### Demographic Characteristics

Demographic characteristics of the study sample are presented in Table 1. Participants were predominantly white (88%, 53/60 participants), but balanced with respect to gender (55% female, 33/60 participants). Age ranged 21 to 70 years, with a mean age of 37 (SEM 2) years. There were no significant differences between education categories for age (*P*=.38), gender (*P*=.17), or race/ethnicity (*P*=.33); however, there were differences in BMI (*P*=.01), income (*P*<.001), and health literacy (*P*<.001) with those in the lower educational category having a higher BMI, and lower income level and health literacy score compared to those in the higher educational category. Differences were found by testing sequence, which was attributed to an unintentional greater random allocation of more participants in the "less than high school/high school" education group being assigned to one of the two sequences.

Completion times for the PP, IMM-1 BEVQ-15 and L-Cat, and IMM-2 BEVQ-15 and L-Cat were approximately three, five, and four minutes, respectively. The paper-based questionnaire time to completion was significantly shorter than both IMM-1 and IMM-2 (both *P*<.001). Time to completion on IMM-2 versus IMM-1 was also significantly different (*P*<.001) with the IMM-1 administration taking longer. There were no differences between education categories for time to completion between the PP (*P*=.69) and the computer-administered versions (IMM-1: *P*=.44; IMM-2: *P*=.73).

**Table 1:** Participant demographic characteristics.^a^

		Less Than High School/High School n=21	Some College/College Degree n=39	Full Sample N=60
**Gender, n (%)**				
	Male	12 (57)	15 (39)	27 (45)
	Female	9 (43)	24 (62)	33 (55)
Age, years		39 (SEM 3)	36 (SEM 2)	37 (SEM 2)
**Race/Ethnicity, n (%)**				
	White	19 (91)	34 (87)	53 (88)
	Black/African-American	2 (10)	1 (3)	3 (5)
	Asian	0	3 (8)	3 (5)
	Other	0	1 (3)	1 (2)
BMI, kg/m^2b^, mean (SEM)		31.8 (2.7)	26.3 (0.7)	28.2 (1.1)
**BMI Category (kg/m** ^**2**^ **), n (%)**				
	Underweight (<18.5)	1 (5)	1 (3)	2 (3)
	Normal (18.5-24.9)	6 (29)	15 (39)	21 (35)
	Overweight (25-29.9)	4 (19)	14 (36)	18 (30)
	Obese (≥30)	10 (48)	9 (23)	19 (32)
Newest Vital Sign (Score^c^)^d^		4.1	5.8	5.2
**Total Annual Household Income, n (%)** ^d^				
	≤$25,000 ^e^	19 (90)	8 (21)	27 (45)
	$25,000-50,000	1 (5)	9 (23)	10 (17)
	≥$50,000	1 (5)	22 (56)	23 (38)

^a^Frequency variables are expressed as n (%), other variables are expressed as mean (SEM).

^b^Significant difference between education groups (*P*=.01).

^c^Scored from 0-6, with 0-1 (high likelihood of limited health literacy), 2-3 (potential limited health literacy), and 4-6 (adequate health literacy) representing the number of correct responses.

^d^Significant difference between education groups (*P*<.001).

^e^Representative of a family of four at or below the current federal income guidelines [41].

### Comparative Validity of the IMM BEVQ-15 and L-Cat

Results from the comparative validity (ie, comparison of the responses from the IMM BEVQ-15 with the validated PP BEVQ-15) assessment of IMM and PP tools for beverage categories are presented in Table 2. Responses from all beverage categories from the IMM-1 and PP versions were correlated (*r*=.34-.98, all *P*<.001), and SSB and total beverage gram and kcal responses were correlated on IMM-1 and PP versions (*r*=.92-.95, all *P*<.001). Between IMM-1 and the PP version, no significant differences in beverage category responses were noted. The mean scores for the PP and IMM-1 L-Cat were 3.5 (SEM 0.2) and 2.4 (SEM 0.2), respectively. The paper-based and IMM-1 L-Cat responses were correlated (*r*=.85, *P*<.001), but mean values were different (*P*<.001).

**Table 2:** Comparative validity of the IMM BEVQ-15: a comparison of the individual beverage category responses from the first IMM administration to the PP BEVQ-15.

		Validity			
Beverage category		IMM-1	Paper	Difference with IMM-1^a^
		Mean (SEM)	Mean (SEM)	Mean (SEM)	Correlation (*r*)
Water (g)		804 (87)	725 (66)	-79 (45)	.866^b^
**100% Fruit juice**					
	g	101 (23)	87 (17)	-14 (14)	.827^b^
	kcal	58 (13)	50 (9)	-8 (8)	.827^b^
**Juice drinks**					
	g	137 (47)	92 (13)	-45 (28)	.817^b^
	kcal	64 (22)	43 (15)	-21 (13)	.817^b^
**Whole milk**					
	g	75 (30)	75 (35)	0 (8)	.981^b^
	kcal	56 (23)	56 (26)	0 (6)	.981^b^
**Reduced-fat milk**					
	g	52 (16)	84 (36)	32 (34)	.339^c^
	kcal	32 (10)	51 (22)	19 (21)	.339^c^
**Fat-free milk**					
	g	68 (19)	83 (19)	15 (11)	.829^b^
	kcal	26 (7)	31 (7)	6 (4)	.829^b^
**Regular soft drinks**					
	g	324 (73)	361 (75)	38 (23)	.951^b^
	kcal	143 (32)	160 (33)	17 (10)	.951^b^
**Diet soft drinks**					
	g	263 (65)	255 (57)	-8 (37)	.828^b^
	kcal	3 (1)	3 (1)	-1 (0)	.828^b^
**Sweet tea**					
	g	211 (54)	186 (52)	-25 (26)	.879^b^
	kcal	68 (17)	60 (17)	-8 (8)	.879^b^
**Sweetened coffee**					
	g	298 (59)	277 (57)	-21 (34)	.823^b^
	kcal	83 (16)	77 (16)	-6 (9)	.831^b^
**Regular coffee/tea**					
	g	168 (48)	246 (60)	78 (39)	.762^b^
	kcal	2 (1)	3 (1)	1 (1)	.758^b^
**Beer**					
	g	101 (32)	98 (32)	-3 (6)	.983^b^
	kcal	35 (11)	34 (11)	-1 (2)	.983^b^
**Liquor**					
	g	17 (8)	19 (10)	3 (4)	.936^b^
	kcal	39 (19)	45 (23)	6 (9)	.936^b^
**Wine**					
	g	23 (6)	18 (6)	-5 (4)	.745^b^
	kcal	16 (4)	13 (4)	-3 (3)	.745^b^
**Energy drinks**					
	g	120 (47)	73 (35)	-47 (30)	.780^b^
	kcal	54 (21)	33 (16)	-21 (13)	.780^b^
**Sugar-sweetened beverage**					
	g	1107 (212)	989 (182)	-177 (72)	.944^b^
	kcal	417 (82)	373 (70)	-44 (28)	.945^b^
**Total beverage**					
	g	2792 (261)	2678 (263)	-115 (106)	.918^b^
	kcal	682 (116)	657 (122)	-25 (42)	.938^b^

^a^Mean difference according to paired sample *t* test; slight difference may be noted from the preceding columns due to rounding, as whole numbers are presented in the table.

^b^ *P*<.001

^c^ *P*=.01

### Test-Retest Reliability of the IMM BEVQ-15 and L-Cat

All beverage category responses on IMM-1 and IMM-2 administrations were correlated (Table 3; *r*=.61-.94, all *P*<.001). SSB and total beverage gram and kcal responses were correlated on both IMM versions (*r*=.73-.96, all *P*<.001). No significant differences in beverage category responses were noted between IMM-1 and IMM-2 with the exception of SSB grams and kcal (*P*=.02 and *P*=.01, respectively) and total beverage kcal (*P*=.01). However, when comparing the responses from the paper and IMM-2 questionnaire administrations on these categories there were no significant differences (mean differences: SSB 63 grams, SEM 87; *P*=.52; SSB 21 kcal, SEM 33; *P*=.48; and total beverage 65 kcal, SEM 49; *P*=.19). The mean L-Cat score on IMM-2 was 2.5 (SEM 0.2)*,* and the IMM L-Cat responses were correlated (*r*=.86, *P*<.001). No significant differences were observed between L-Cat responses on the IMM-1 and IMM-2 questionnaires (*P*=.72); however, differences were noted in IMM-2 and PP L-Cat responses (*P*<.001).

**Table 3:** Reproducibility of the IMM BEVQ-15: Comparison of the first and second IMM administrations.

		Reliability			
Beverage Category		IMM-1	IMM-2	Difference with IMM-1^a^
		Mean (SEM)	Mean (SEM)	Mean (SEM)	Correlation (*r*)

Water (g)		804 (87)	756 (76)	-47 (62)	.721^b^
**100% Fruit juice**					
	g	101 (23)	76 (17)	-24 (18)	.666^b^
	kcal	58 (13)	44 (10)	-13 (10)	.665^b^
**Juice drinks**					
	g	137 (47)	134 (48)	-3 (23)	.888^b^
	kcal	64 (22)	63 (22)	-1 (10)	.888^b^
**Whole milk**					
	g	75 (30)	54 (26)	-20 (13)	.898^b^
	kcal	56 (23)	41 (20)	-15 (10)	.898^b^
**Reduced-fat milk**					
	g	52 (16)	33 (10)	-19 (10)	.807^b^
	kcal	32 (10)	20 (6)	-12 (6)	.807^b^
**Fat-free milk**					
	g	68 (19)	74 (18)	6 (11)	.811^b^
	kcal	26 (7)	28 (7)	2 (4)	.811^b^
**Regular soft drinks**					
	g	324 (73)	289(69)	-35 (35)	.881^b^
	kcal	143 (32)	128 (31)	-15 (15)	.881^b^
**Diet soft drinks**					
	g	263 (65)	246 (63)	-16 (42)	.784^b^
	kcal	3 (1)	3 (1)	0 (0)	.784^b^
**Sweet tea**					
	g	211 (54)	172 (53)	-39 (31)	.834^b^
	kcal	68 (17)	55 (17)	-12 (10)	.834^b^
**Sweetened coffee**					
	g	298 (59)	254 (52)	-44 (45)	.676^b^
	kcal	83 (16)	71 (14)	-13 (12)	.689^b^
**Regular coffee/tea**					
	g	168 (48)	199 (51)	31 (44)	.616^b^
	kcal	2 (1)	2 (1)	0 (1)	.610^b^
**Beer**					
	g	101 (32)	121 (42)	19 (24)	.830^b^
	kcal	35 (11)	42 (15)	7 (8)	.830^b^
**Liquor**					
	g	17 (8)	19 (8)	3 (3)	.944^b^
	kcal	39 (19)	45 (19)	6 (6)	.944^b^
**Wine**					
	g	23 (6)	24 (6)	1 (4)	.827^b^
	kcal	16 (4)	17 (4)	1 (3)	.827^b^
**Energy drinks**					
	g	120 (47)	78 (34)	-42 (30)	.771^b^
	kcal	54 (21)	35 (14)	-19 (14)	.771^b^
**Sugar-sweetened beverage**					
	g	1107 (212)	927(189)	-180 (71^c^)	.944^b^
	kcal	417 (82)	351 (75)	-65 (26^d^)	.948^b^
**Total beverage**					
	g	2792 (261)	2755 (296)	-38 (207)	.731^b^
	kcal	682 (116)	592 (107)	-91 (34 ^d^)	.955^b^

^a^Mean difference according to paired sample *t* test; slight difference may be noted from the preceding columns due to rounding, as whole numbers are presented in the table.

^b^Significant correlations (*P*<.001)

^c^Significant difference between IMM-2 and IMM-1 (*P*=.02).

^d^Significant difference between IMM-2 and IMM-1 (*P*=.01).

### Comparative Validity and Reliability Within Educational Categories

**Figure 2:**
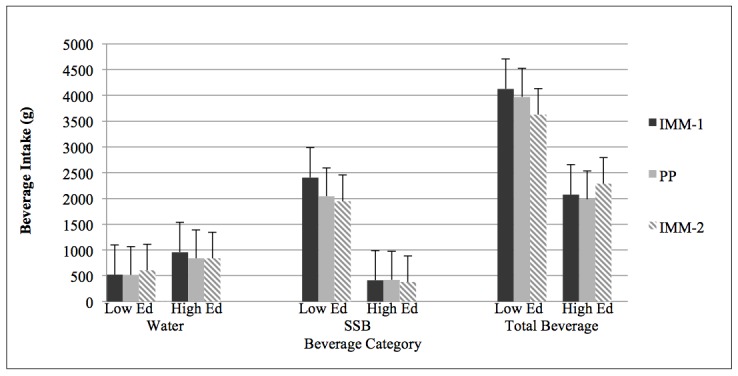
Beverage intake in grams for education categories. The following abbreviations mean: Low Ed=Less than high school/high school; High Ed=Some college/college degree; SSB=Sugar-sweetened beverages; IMM=Interactive Multi-media, 2 separate administrations; and PP=Paper and Pencil version. The "Water" "Low Ed" solid black and solid grey bars and the "Total Beverage" "Low Ed" grey-striped bar show a significant difference from "High Ed" group (*P*=.02). The "SSB" "Low Ed" solid black, solid grey, and grey-striped bars and "Total Beverage" "Low Ed" solid black and solid grey bars show a significant difference from "High Ed" group (*P*<.001).

**Figure 3:**
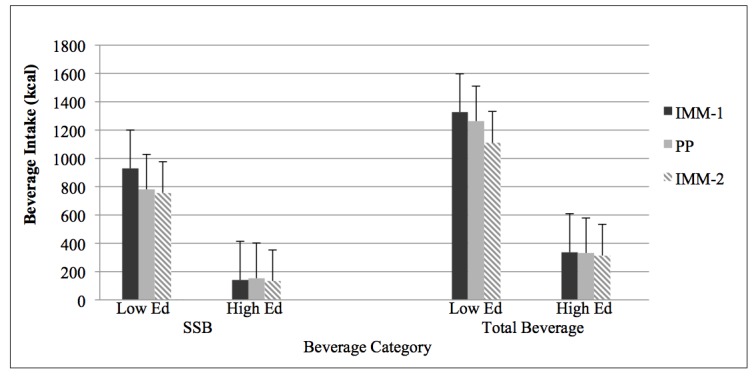
Beverage intake in calories (kcal) for education categories. The following abbreviations mean: Low Ed=Less than high school/high school, High Ed=Some college/college degree; SSB=Sugar-sweetened beverages; IMM=Interactive Multi-media, two separate administrations; and PP=Paper and Pencil version. The six "Low Ed" solid black, solid grey, and grey-striped bars show a significant difference from "High Ed" group (*P*<.001).

**Figure 4:**
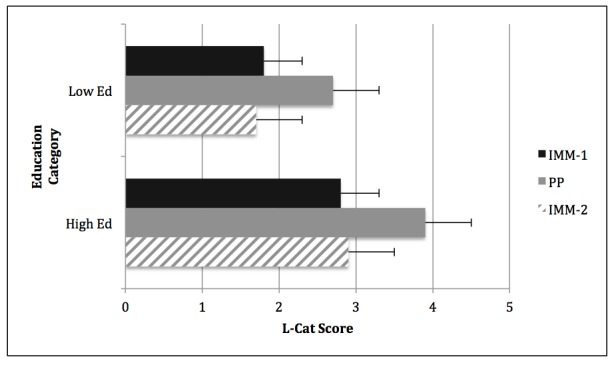
Comparison of education group responses on the Stanford Leisure-Time Activity Categorical Item (L-Cat). The following abbreviations mean: Low Ed=Less than high school/high school; High Ed=Some college/college degree; IMM=Interactive Multi-media, two separate administrations; and PP=Paper and Pencil version. The "Low Ed" top black solid bar shows the significant difference from the "High Ed" group (*P*=.008). The "Low Ed" center grey solid bar shows the significant difference from the "High Ed" group (*P*=.002). The "Low Ed" bottom grey-striped bar shows the significant difference from the "High Ed" group (*P*=.001). The "Low Ed" and "High Ed" center solid grey bars show the significant difference from "IMM-1" and "IMM-2" (both *P*<.001).

Figures 2-4 show the results of the major beverage outcomes (water, SSB, total beverage intake) and L-Cat according to educational level. The IMM version of the beverage questionnaire demonstrated comparative validity across the major beverage outcomes with the exception of water intake in the "some college/college degree" participants (mean difference between PP and IMM-1 119, SEM 59; *P*=.048) with correlation coefficients ranging from .76-.95 (all *P*<.001). However, responses to the L-Cat were significantly higher with the PP version in both educational groups (mean differences between PP and IMM-1 in less than high school/high school 0.9, SEM 0.2; and in some college/college degree 1.1, SEM 0.1; both *P*<.001).

No differences were noted in repeated IMM responses for beverage intake or physical activity. Differences were noted in the repeated IMM responses from the "less than high school/high school" participants for SSB grams and kcal (both *P*=.02) and total beverage energy (*P*=.03), with lower intake reported on the second administration. However, pair samples *t* tests results revealed no significant differences between the PP and IMM-2 tools (mean differences–SSB 99 grams, SEM 245; *P*=.69; SSB 24 kcal, SEM 92; *P*=.79; total beverage 152 kcal, SEM 134; *P*=.27). There were no significant differences on L-Cat responses in the "less than high school/high school" participants (*P*=.67).

### Comparison of Lower and Higher Educational Groups

Differences in water consumption between education categories on the first IMM and PP questionnaire administrations were significant (both *P*=.02), but not on the second IMM (*P*=.14), with the "less than high school/high school" participants reporting significantly lower water consumption than the "some college/college degree" participants (Figure 2). Daily SSB (grams and kcal) and total beverage consumption (grams and kcal) were different between education categories at each questionnaire administration (IMM-1, IMM-2, PP) (Figures 2 and 3). Differences between education categories were noted at each L-Cat administration (IMM-1: *P*=.008; IMM-2: *P*=.001; PP: *P*=.002) (Figure 4).

### User Feedback

Of the 60 participants, 59 completed the feedback survey. *Major themes* were as follows. Most believed that the IMM BEVQ-15 was "easy"(mean ordered-response rating 1.2, SEM 0.1; n=54), that it was "clear" or "straightforward"(mean 1.4, SEM 0.1; n=30), and that it covered beverages consistent with their usual intake habits (mean 1.1, SEM 0.0; n=56). Most also reported that the computerized L-Cat was "easy" (mean 1.2, SEM 0.1; n=49), and that they were able to identify a physical activity statement relating to their lifestyle with the L-Cat (mean 1.1, SEM 0.1; n=49). *Minor themes* were that the graphics, images, and voiceover made completing the questionnaires "easy" (mean 1.4, SEM 0.1; n=15), and that nothing needed to be changed in the IMM BEVQ-15 (mean 1.0, SEM 0.1; n=22). Many participants reported that the IMM L-Cat assessment was "clear" or "straightforward" (mean 1.6, SEM 0.1; n=23), and recommended changing nothing about the IMM L-Cat (mean 1.0, SEM 0.1; n=23). Eighteen participants of 60 (30%) suggested that the speed of the narrated text be increased on both the IMM BEVQ-15 and L-Cat. Only two participants (one in each education category) reported the computerized BEVQ-15 as being "hard."

##  Discussion

### Comparative Validity and Test-Reliability of the IMM BEVQ-15 and L-Cat

With the exception of reduced-fat milk, all correlations were greater than .74 (Table 2) and this can be considered superior to other validation studies where typical *r* values range from .4 to .7 [3], and consistent with initial BEVQ testing [36]. The lower correlation coefficient for reduced-fat milk may be due to participants not being familiar with the form of milk they consume. Some participants may not be the primary food shoppers in their home and not read product packaging prior to consumption; they may not know if the milk they choose to consume is skim, 1%, 2%, or whole milk, and thus choose an option arbitrarily on the questionnaire. The differences between PP and IMM-1 for total beverage intake were 25 kcal, and between PP and IMM-2, while higher at approximately 90 kcal, were not statistically significant. However, we recognize that a 90 kcal difference can be clinically significant at the individual level. Compared to the original BEVQ validation studies, the present differences are higher than what was observed for SSB and total beverage kcal intake. The lower mean age, smaller sample size, and the beverage intake patterns of the "less than high school/high school" education group may have contributed to differences across studies. Future investigations including a larger sample size could provide greater insight into this possibility.

While no significant differences in beverage category responses were noted between IMM-1 and the PP version, mean values were different on the PP and both IMM physical activity items (both *P*<.001). Computer-based tools can be perceived as more private and less intimidating [7]. If this were so in the present study, the IMM L-Cat responses may be more reflective of actual physical activity levels. Participants may have felt more comfortable reporting a less socially desirable level of physical activity on the computerized assessment versus on the paper-based tool since, when taking the paper version, responses were immediately observable to study personnel, while computerized responses would be accessed following the participant's departure. Overall, the findings of this pilot investigation indicate that the IMM BEVQ-15 is a valid measure of beverage intake when compared to the PP version. Further research is warranted to assess the comparative validity of the IMM L-Cat.

Differences were observed between SSB grams and kcal and total beverage intake (*P*=.02 and *P*=.01, respectively) and total beverage kcal (*P*=.01) between IMM-1 and IMM-2; however, no differences were apparent when comparing these responses on the paper and IMM-2 responses. Thus, the lower responses for the reported usual intake on the second IMM administration were closer to that reported in the PP tool. This may be attributed to a familiarity effect as observed in other computerized assessments [42-44]. In a trial investigating how test mode may impact assessment outcomes, content familiarity and not computer familiarity, gender, or competitiveness positively influenced test performance [16].

Food frequency questionnaires are considered reliable with correlations ranging from .5 to .7 [3,45,46], and many of the coefficients observed for reliability testing of the IMM questionnaires are within or exceed this rage. Thus, the IMM BEVQ-15 and L-Cat can be considered reliable measures of habitual beverage intake and physical activity patterns.

### Comparative Validity and Reliability Within Educational Categories

With the exception of water intake in the "some college/college degree" group, the IMM version of the BEVQ-15 demonstrated comparative validity across the major beverage categories. As depicted in Figure 4, both educational groups responded significantly higher on the paper version versus the computerized version of the L-Cat. Since the layout and appearance of computerized surveys can impact participant responses [16,17], differences between the IMM and PP modes of assessment may have occurred in the present investigation. Although lower intakes of SSB grams, kcal, and total beverage kcal were reported on IMM-2 compared to IMM-1 from the "less than high school/high school" participants, no differences were observed between the paper and IMM-2 tools. These results may be attributed to participants being more familiar with the IMM version at the second administration, as stated earlier. Participants potentially had a greater awareness of the upcoming beverage categories within the IMM tool, and thus were able to answer each question more accurately, better reflecting their usual consumption habits.

### Comparison of Lower and Higher Educational Groups

In the present investigation, the "less than high school/high school" participants reported significantly lower water consumption than the "some college/college degree" participants (Figure 2). Similarly, the National Health and Nutrition Examination Surveys from 1999-2006 revealed that adults with higher education attainment had a higher plain water intake [47]. In addition, daily grams and kcal were different between educational categories at each questionnaire administration (Figures 2 and 3). Consistent with prior research addressing the influence of educational level and health literacy on beverage consumption patterns [48-50], participants in the lower educational category consumed significantly more total beverage and SSB (grams, kcal). This is noteworthy since excessive SSB consumption has been related to the development of some chronic diseases [51-54]. Similar to the discrepancies observed between physical activity engagement and education attainment in a recent report from the American Heart Association Statistic Committee and Stroke Statistics Subcommittee [55], the "less than high school/high school" category reported lower physical activity engagement than those with "some college/college degree" (Figure 4). However, neither group reported a level of physical activity that met current guidelines [56], exemplifying the need for continued efforts to promote the benefits of regular participation in physical activity.

### Usability and User Feedback

Although no differences in completion time were observed between educational categories, the IMM-1 administration took significantly more time to complete versus IMM-2. This is possibly due to unfamiliarity with page-to-page navigation and questionnaire content at the first IMM administration [16].

Participants found the IMM questionnaires easy to use, and that they "fit" their usual beverage intake and leisure-time activity habits. Our results are comparable to others who have reported positive feedback with IMM delivery of nutrition education and dietary and physical activity assessments [7,8,12,14], suggesting acceptability and promise for the use of computer-administered surveys in future research. One area for potential improvement in the IMM tools is the speed of narrated text. Analogous to prior research [7], approximately one-third (n=18) of participants suggested that the speed of the voiceover be increased. The present study received positive feedback overall; however, improvements can be made with the IMM itself (eg, touch screens) [7], which may further increase ease of use and administration.

### Limitations

Strengths of this pilot investigation include the random assignment of participants to study session sequences, the novel method to assess dietary intake and physical activity engagement, and the inclusion of participants with lower and higher educational attainment levels. However, several limitations are recognized. The short duration between participant sessions could have caused acclimatization to the questionnaires or participants to be more aware of their beverage intake patterns, thus biasing their responses. Subsequent trials should consider both familiarity with computers and questionnaire content [16,57,58] during participant screening and longer intervals between study sessions. Second, we used validated paper versions of the BEVQ-15 and L-Cat as our comparative criterion; however, self-reported diet and physical activity assessments may not reflect an individual's true intake [1,3,59] or physical activity engagement [60]. In addition, some consider paper versions of computerized surveys to be anything but a "gold standard" when assessing computerized versions of similar assessments [6]. Future studies should not only use validated paper forms of computerized questionnaires, but multiple modes of dietary and physical activity assessment (eg, 24 hour recalls and doubly labeled water) for the greatest degree of accuracy. Although different circumstances may impact beverage intake from day to day (eg, illness, activity level, social events), we do not believe this influenced our overall findings, since the BEVQ-15 has been found valid in estimating group habitual beverage intake [18]. Another potential limitation of the present investigation is the limited racial representation and small sample size. Subsequent larger-scale investigations should include a more diverse sample in terms of race/ethnicity and educational attainment.

### Conclusions

There is a need for reliable and valid dietary and physical activity assessment tools that are brief and easily administered [61]. As many as 20% of American adults read at a fifth-grade level or less [62,63], and health literacy is thought to better predict a person's health than ethnicity, employment status, age, income, and education level [64]. Using computer-based assessments can overcome some common barriers preventing the collection of complete dietary data [13], particularly in populations with lower educational achievement [8]. Interactive multimedia versions of dietary and physical activity questionnaires have the potential to decrease participant and study personnel burden, allowing for high quality data to be collected and analyzed [6-8,10,13,14]. Further, computerized assessments could be advantageous for large epidemiological studies [6] as they may reduce costs [8] by streamlining data collection and analysis [4,6-8,13,14]. Overall, the results of the present investigation show that the IMM BEVQ-15 may be used to evaluate habitual beverage intake; although, familiarizing participants with the software prior to data collection may assist in obtaining more accurate data. Respondents may have answered differently on the IMM L-Cat due to computerized tools being considered more confidential and less intimidating [7]. Further research is necessary to fully evaluate the validity of the IMM L-Cat due to its consistency between IMM measures, but differences from the PP version. Future investigations are warranted to include more participants from racially diverse and hard-to-reach audiences (ie, low educational and health literacy levels), develop assessment tools that may be administered to both younger and older individuals (eg, children, adolescents, seniors), and utilize contemporary technological features to further reduce participant burden.

## Abbreviations

BEVQ: beverage intake questionnaire
BEVQ-15: beverage intake questionnaire
BMI: body mass index
IMM: interactive multimedia
L-Cat: Stanford Leisure-Time Activity Categorical Item
NVS: Newest Vital Sign
PP: paper-administered
SSB: sugar-sweetened beverage

## References

[1] Monsen ER, Horn L, Van Horn L, Johnson RK, Yon BA, Hankin JH (2008). Dietary Assessment and Validation. Research: successful approaches.

[2] Shephard RJ (2003). Limits to the measurement of habitual physical activity by questionnaires. Br J Sports Med.

[3] Thompson FE, Subar AF (2013). Chapter 1 - Dietary Assessment Methodology.

[4] Ekman A, Dickman PW, Klint A, Weiderpass E, Litton JE (2006). Feasibility of using web-based questionnaires in large population-based epidemiological studies. Eur J Epidemiol.

[5] Ekman A, Klint A, Dickman PW, Adami HO, Litton JE (2007). Optimizing the design of web-based questionnaires--experience from a population-based study among 50,000 women. Eur J Epidemiol.

[6] Ekman A, Litton JE (2007). New times, new needs; e-epidemiology. Eur J Epidemiol.

[7] Jantz C, Anderson J, Gould SM (2002). Using computer-based assessments to evaluate interactive multimedia nutrition education among low-income predominantly Hispanic participants. J Nutr Educ Behav.

[8] Zoellner J, Anderson J, Gould SM (2005). Comparative validation of a bilingual interactive multimedia dietary assessment tool. J Am Diet Assoc.

[9] INTERNET USAGE STATISTICS The Internet Big Picture.

[10] Bälter KA, Bälter O, Fondell E, Lagerros YT (2005). Web-based and mailed questionnaires: a comparison of response rates and compliance. Epidemiology.

[11] Noyes JM, Garland KJ (2008). Computer- vs. paper-based tasks: are they equivalent?. Ergonomics.

[12] Bonn SE, Trolle Lagerros Y, Christensen SE, Möller E, Wright A, Sjölander A, Bälter K (2012). Active-Q: validation of the web-based physical activity questionnaire using doubly labeled water. J Med Internet Res.

[13] Kohlmeier L, Mendez M, McDuffie J, Miller M (1997). Computer-assisted self-interviewing: a multimedia approach to dietary assessment. Am J Clin Nutr.

[14] Touvier M, Méjean C, Kesse-Guyot E, Pollet C, Malon A, Castetbon K, Hercberg S (2010). Comparison between web-based and paper versions of a self-administered anthropometric questionnaire. Eur J Epidemiol.

[15] Illner AK, Freisling H, Boeing H, Huybrechts I, Crispim SP, Slimani N (2012). Review and evaluation of innovative technologies for measuring diet in nutritional epidemiology. Int J Epidemiol.

[16] Clariana R, Wallace P (2002). Paper-based versus computer-based assessment: key factors associated with the test mode effect. Br J Educ Technol.

[17] Wang H, Shin CD Test, Measurement & Research Services Bulletin.

[18] Hedrick VE, Savla J, Comber DL, Flack KD, Estabrooks PA, Nsiah-Kumi PA, Ortmeier S, Davy BM (2012). Development of a brief questionnaire to assess habitual beverage intake (BEVQ-15): sugar-sweetened beverages and total beverage energy intake. J Acad Nutr Diet.

[19] Kiernan M, Schoffman DE, Lee K, Brown SEM, Fair JM, Perri MG, Haskell WL (2013). The Stanford Leisure-Time Activity Categorical Item (L-Cat): a single categorical item sensitive to physical activity changes in overweight/obese women. Int J Obes (Lond).

[20] Oenema A, Brug J, Lechner L (2001). Web-based tailored nutrition education: results of a randomized controlled trial. Health Educ Res.

[21] Riley WT, Beasley J, Sowell A, Behar A (2007). Effects of a Web-based food portion training program on food portion estimation. J Nutr Educ Behav.

[22] Baranowski T, Islam N, Baranowski J, Cullen KW, Myres D, Marsh T, de MC (2002). The food intake recording software system is valid among fourth-grade children. J Am Diet Assoc.

[23] Beasley JM, Davis A, Riley WT (2009). Evaluation of a web-based, pictorial diet history questionnaire. Public Health Nutr.

[24] Labonté MÈ, Cyr A, Baril-Gravel L, Royer MM, Lamarche B (2012). Validity and reproducibility of a web-based, self-administered food frequency questionnaire. Eur J Clin Nutr.

[25] Matthys C, Pynaert I, De Keyzer W, De Henauw S (2007). Validity and reproducibility of an adolescent web-based food frequency questionnaire. J Am Diet Assoc.

[26] Probst YC, Tapsell LC (2005). Overview of computerized dietary assessment programs for research and practice in nutrition education. J Nutr Educ Behav.

[27] Raper N, Perloff B, Ingwersen L, Steinfeldt L, Anand J (2004). An overview of USEMA's Dietary Intake Data System. Journal of Food Composition and Analysis.

[28] Vandelanotte C, De Bourdeaudhuij I, Brug J (2004). Acceptability and feasibility of an interactive computer-tailored fat intake intervention in Belgium. Health Promot Int.

[29] De Vera MA, Ratzlaff C, Doerfling P, Kopec J (2010). Reliability and validity of an internet-based questionnaire measuring lifetime physical activity. Am J Epidemiol.

[30] Namba H, Yamaguchi Y, Yamada Y, Tokushima S, Hatamoto Y, Sagayama H, Kimura M, Higaki Y, Tanaka H (2012). Validation of Web-based physical activity measurement systems using doubly labeled water. J Med Internet Res.

[31] National Cancer Institute (2013). Applied Research: Cancer Control and Population Sciences.

[32] Subar AF, Kirkpatrick SI, Mittl B, Zimmerman TP, Thompson FE, Bingley C, Willis G, Islam NG, Baranowski T, McNutt S, Potischman N (2012). The Automated Self-Administered 24-hour dietary recall (ASA24): a resource for researchers, clinicians, and educators from the National Cancer Institute. J Acad Nutr Diet.

[33] Kant AK, Graubard BI (2007). Secular trends in the association of socio-economic position with self-reported dietary attributes and biomarkers in the US population: National Health and Nutrition Examination Survey (NHANES) 1971-1975 to NHANES 1999-2002. Public Health Nutr.

[34] Weiss BD, Mays MZ, Martz W, Castro KM, DeWalt DA, Pignone MP, Mockbee J, Hale FA (2005). Quick assessment of literacy in primary care: the newest vital sign. Ann Fam Med.

[35] Comber DL, Respress V, Estabrooks P, Almeida F, Davy BM (2009). Abstract presented at: Obesity Society Annual Meeting; October 24-28; Washington DC.

[36] Hedrick VE, Comber DL, Estabrooks PA, Savla J, Davy BM (2010). The beverage intake questionnaire: determining initial validity and reliability. J Am Diet Assoc.

[37] Taylor-Piliae RE, Fair JM, Haskell WL, Varady AN, Iribarren C, Hlatky MA, Go AS, Fortmann SP (2010). Validation of the Stanford Brief Activity Survey: examining psychological factors and physical activity levels in older adults. J Phys Act Health.

[38] Taylor-Piliae RE, Haskell WL, Iribarren C, Norton LC, Mahbouba MH, Fair JM, Hlatky MA, Go AS, Fortmann SP (2007). Clinical utility of the Stanford brief activity survey in men and women with early-onset coronary artery disease. J Cardiopulm Rehabil Prev.

[39] Taylor-Piliae RE, Norton LC, Haskell WL, Mahbouda MH, Fair JM, Iribarren C, Hlatky MA, Go AS, Fortmann SP (2006). Validation of a new brief physical activity survey among men and women aged 60-69 years. Am J Epidemiol.

[40] Bogdan R, Biklen SK (2007). Qualitative research for education: an introduction to theories and methods.

[41] Health and Human Services Department (2013). Federal Register:The Daily Journal of the United States Government.

[42] Bennett RE, Braswell J, Oranje A, Sandene B, Kaplan B, Yan F The Journal of Technology, Learning, and Assessment.

[43] Pomplun M, Frey S, Becker DF (2002). The Score Equivalence of Paper-and-Pencil and Computerized Versions of a Speeded Test of Reading Comprehension. Educational and Psychological Measurement.

[44] Pomplun M, Ritchie T, Custer M (2006). Factors in Paper-and-Pencil and Computer Reading Score Differences at the Primary Grades. Educational Assessment.

[45] Willett W (2012). Nutritional Epidemiology (Monographs in Epidemiology and Biostatistics).

[46] Willett WC (1994). Future directions in the development of food-frequency questionnaires. Am J Clin Nutr.

[47] Kant AK, Graubard BI, Atchison EA (2009). Intakes of plain water, moisture in foods and beverages, and total water in the adult US population--nutritional, meal pattern, and body weight correlates: National Health and Nutrition Examination Surveys 1999-2006. Am J Clin Nutr.

[48] Ferguson K, Davy B, Zoellner J, You W (2011). Digital Library and Archives.

[49] Zoellner J, You W, Connell C, Smith-Ray RL, Allen K, Tucker KL, Davy BM, Estabrooks P (2011). Health literacy is associated with healthy eating index scores and sugar-sweetened beverage intake: findings from the rural Lower Mississippi Delta. J Am Diet Assoc.

[50] Thompson FE, McNeel TS, Dowling EC, Midthune D, Morrissette M, Zeruto CA (2009). Interrelationships of added sugars intake, socioeconomic status, and race/ethnicity in adults in the United States: National Health Interview Survey, 2005. J Am Diet Assoc.

[51] de Koning L, Malik VS, Kellogg MD, Rimm EB, Willett WC, Hu FB (2012). Sweetened beverage consumption, incident coronary heart disease, and biomarkers of risk in men. Circulation.

[52] de Koning L, Malik VS, Rimm EB, Willett WC, Hu FB (2011). Sugar-sweetened and artificially sweetened beverage consumption and risk of type 2 diabetes in men. Am J Clin Nutr.

[53] Malik VS, Popkin BM, Bray GA, Després JP, Hu FB (2010). Sugar-sweetened beverages, obesity, type 2 diabetes mellitus, and cardiovascular disease risk. Circulation.

[54] Malik VS, Popkin BM, Bray GA, Després JP, Willett WC, Hu FB (2010). Sugar-sweetened beverages and risk of metabolic syndrome and type 2 diabetes: a meta-analysis. Diabetes Care.

[55] Go AS, Mozaffarian D, Roger VL, Benjamin EJ, Berry JD, Borden WB, Bravata DM, Dai S, Ford ES, Fox CS, Franco S, Fullerton HJ, Gillespie C, Hailpern SM, Heit JA, Howard VJ, Huffman MD, Kissela BM, Kittner SJ, Lackland DT, Lichtman JH, Lisabeth LD, Magid D, Marcus GM, Marelli A, Matchar DB, McGuire DK, Mohler ER, Moy CS, Mussolino ME, Nichol G, Paynter NP, Schreiner PJ, Sorlie PD, Stein J, Turan TN, Virani SS, Wong ND, Woo D, Turner MB, American Heart Association Statistics CommitteeStroke Statistics Subcommittee (2013). Heart disease and stroke statistics--2013 update: a report from the American Heart Association. Circulation.

[56] United States Department of Health and Human Services (2008). U.S. Department of Health and Human Services.

[57] Wallace P, Clariana R (2000). Journal of Information Systems Education.

[58] Watson DB (2001). Key factors affecting conceptual gains from CAL materials. Br J Educ Technol.

[59] Schoeller DA, Thomas D, Archer E, Heymsfield SB, Blair SN, Goran MI, Hill JO, Atkinson RL, Corkey BE, Foreyt J, Dhurandhar NV, Kral JG, Hall KD, Hansen BC, Heitmann BL, Ravussin E, Allison DB (2013). Self-report-based estimates of energy intake offer an inadequate basis for scientific conclusions. Am J Clin Nutr.

[60] Taber DR, Stevens J, Murray DM, Elder JP, Webber LS, Jobe JB, Lytle LA (2009). The effect of a physical activity intervention on bias in self-reported activity. Ann Epidemiol.

[61] Yaroch AL, Tooze J, Thompson FE, Blanck HM, Thompson OM, Colón-Ramos U, Shaikh AR, McNutt S, Nebeling LC (2012). Evaluation of three short dietary instruments to assess fruit and vegetable intake: the National Cancer Institute's food attitudes and behaviors survey. J Acad Nutr Diet.

[62] Kirsch IS (1993). Adult Literacy in America: A First Look at the Results of the National Adult Literacy Survey.

[63] Kirsch I (2001). The International Adult Literacy Survey (IALS): Understanding What Was Measured.

[64] Parker RM, Williams MV, Baker DW, Weiss BD, Davis TC, Doak CC, Doak LG, Heln K, Meade CD, Nurss J, Schwartzberg JG, Somers SA (1999). Health literacy: report of the Council on Scientific Affairs. Ad Hoc Committee on Health Literacy for the Council on Scientific Affairs, American Medical Association. JAMA.

